# Myocardial Ablation of G Protein–Coupled Receptor Kinase 2 (GRK2) Decreases Ischemia/Reperfusion Injury through an Anti-Intrinsic Apoptotic Pathway

**DOI:** 10.1371/journal.pone.0066234

**Published:** 2013-06-21

**Authors:** Qian Fan, Mai Chen, Lin Zuo, Xiying Shang, Maggie Z. Huang, Michele Ciccarelli, Philip Raake, Henriette Brinks, Kurt J. Chuprun, Gerald W. Dorn, Walter J. Koch, Erhe Gao

**Affiliations:** 1 Center for Translational Medicine, Temple University School of Medicine, Philadelphia, Pennsylvania, United States of America; 2 The Center for Pharmacogenomics, Department of Medicine, Washington University School of Medicine, St. Louis, Missouri, United States of America; 3 Chaoyang Hospital, Capital Medical University, Beijing, China; 4 Xijing Hospital, The Fourth Military Medical University, Xian, China; Brigham & Women’s Hospital - Harvard Medical School, United States of America

## Abstract

Studies from our lab have shown that decreasing myocardial G protein–coupled receptor kinase 2 (GRK2) activity and expression can prevent heart failure progression after myocardial infarction. Since GRK2 appears to also act as a pro-death kinase in myocytes, we investigated the effect of cardiomyocyte-specific GRK2 ablation on the acute response to cardiac ischemia/reperfusion (I/R) injury. To do this we utilized two independent lines of GRK2 knockout (KO) mice where the GRK2 gene was deleted in only cardiomyocytes either constitutively at birth or in an inducible manner that occurred in adult mice prior to I/R. These GRK2 KO mice and appropriate control mice were subjected to a sham procedure or 30 min of myocardial ischemia via coronary artery ligation followed by 24 hrs reperfusion. Echocardiography and hemodynamic measurements showed significantly improved post-I/R cardiac function in both GRK2 KO lines, which correlated with smaller infarct sizes in GRK2 KO mice compared to controls. Moreover, there was significantly less TUNEL positive myocytes, less caspase-3, and -9 but not caspase-8 activities in GRK2 KO mice compared to control mice after I/R injury. Of note, we found that lowering cardiac GRK2 expression was associated with significantly lower cytosolic cytochrome C levels in both lines of GRK2 KO mice after I/R compared to corresponding control animals. Mechanistically, the anti-apoptotic effects of lowering GRK2 expression were accompanied by increased levels of Bcl-2, Bcl-xl, and increased activation of Akt after I/R injury. These findings were reproduced in vitro in cultured cardiomyocytes and GRK2 mRNA silencing. Therefore, lowering GRK2 expression in cardiomyocytes limits I/R-induced injury and improves post-ischemia recovery by decreasing myocyte apoptosis at least partially via Akt/Bcl-2 mediated mitochondrial protection and implicates mitochondrial-dependent actions, solidifying GRK2 as a pro-death kinase in the heart.

## Introduction

The myocyte death that follows acute myocardial ischemia and subsequent reperfusion (I/R) injury is a major factor contributing to high mortality and morbidity in ischemic heart disease. Death of myocytes after I/R injury can be due to autophagy, necrosis, or apoptosis [1]. Apoptotic cell death is primarily orchestrated by caspases, a group of aspartate-specific cysteine proteases which reside in the cytosol as inactive proforms in healthy cells [2], [3]. The activation of caspases is controlled by two distinct pathways: the death receptor (extrinsic) pathway and the mitochondrial (intrinsic) pathway [4], [5]. The “extrinsic” pathway is triggered by the binding of ligands, such as tumor necrosis factor and Fas, to their cognate receptors to induce receptor clustering and the formation of a death-inducing signaling complex (DISC) [6], [7]. This complex recruits multiple procaspase-8 molecules via an adaptor molecule FADD (Fas-associated death domain protein), resulting in the activation of caspase-8 and downstream caspase-3 [6]. The “intrinsic” pathway utilizes mitochondria to produce cell death through opening of the mitochondrial permeability transition pore (mPTP), triggering the sudden release of cytochrome C and other proteins from the intermembrane space of mitochondria into the cytosol [8]. Released cytochrome C facilitates formation of the “apoptosome” complex, which results in caspase-9 activation and subsequent activation of caspase-3, the final effector of apoptosis [5], [9].

In myocytes, the “intrinsic” pathway is activated by a variety of cellular stimuli such as oxidative stress and hypoxia, which occurs after ischemic injury [10], [11]. Several studies have shown that following I/R, cardiomyocyte apoptosis is controlled, at least in part, by Bcl-2 family members [1], [12]. Among the Bcl-2 family, Bcl-xL and Bcl-2 are known to be anti-apoptotic, whereas Bax, Bak, and Bad are pro-apoptotic [1], [13], [14]. Bad, through its Bcl-2 homology-3 domain, mediates its death-promoting activity through the binding of Bcl-xL. However, phosphorylation of Bad by pro-survival kinases, such as Akt, leads to the release of both Bcl-xL and Bcl-2 [15], [16]. Phosphorylation of Bax retains it in the cytoplasm and prevents translocation to mitochondria [17]–[19].

G protein–coupled receptor (GPCR) kinase 2 (GRK2), a critical regulator of cardiac GPCRs such as β-adrenergic receptors (βARs) has been also shown to be a key regulator of cardiac regulation and contractile function [20]. GRK2 is up- regulated in both acute and chronic heart failure (HF) [21], [22]. In fact, studies have shown that after myocardial ischemic injury, GRK2 up-regulation is an early event and is ultimately responsible for crippling the myocardial βAR system [23]. Recent studies from our lab have shown that silencing myocardial GRK2 expression [22] or preventing GRK2 activity (with a peptide inhibitor) [24] can prevent or rescue HF progression after myocardial infarction. Further, we have recently shown that when GRK2 expression is increased in the heart, as seen with cardiac stress, significantly more severe ischemic injury is seen after I/R injury [25]. The mechanism by which GRK2 promotes cell death in the heart is not fully understood as it appears to be associated with increased apoptosis due to reduced activation of the Akt and subsequent nitric oxide production [25].

In the present study, we investigated the direct role of GRK2 in the regulation of myocardial apoptosis by using cardiac-specific GRK2 knockout (KO) mice and an in vivo I/R injury model. Importantly, we not only confirmed our previous findings that GRK2 activity is associated with pro-death signaling after stress that can negatively impact post-I/R function, we uncovered novel information concerning potential signaling mechanisms including implications for mitochondrial-dependent effects. These data support inhibition of GRK2 as a therapeutic strategy for cardioprotection.

## Methods

### Experimental Animals

Mice bearing floxed GRK2 alleles (GRK2 fl/fl) along with cardiac-specific transgenic mice harboring Cre recombinase either constitutively via the α-myosin heavy chain (αMHC) promoter or tamoxifin (Tmx)-inducible via Cre fused to mutant estrogen receptors (MerCreMer, MCM) have been previously described and utilized by our laboratory [22], [26], [27]. GRK2fl/fl mice were bred with these Cre Transgenic mice and resultant mice were defined as GRK2KO (constitutive) or GRK2iKO (inducible) mice. These mice were used for all experiments along with appropriate control, wild-type (WT) mice (including GRK2 fl/fl and MMC mice). All mice were in the C57/B6 genetic background and all animal procedures and experiments were carried out according to National Institutes of Health Guidelines on the Use of Laboratory Animals and approved by the Animal Care and Use Committee of Thomas Jefferson University and Temple University.

### Experimental Groups and Protocol

GRK2KO, GRK2iKO along with appropriate and WT control mice were subjected to either a sham procedure or 30 min myocardial ischemia via coronary artery ligation followed by reperfusion to induce I/R injury as described [28]. At 24 hrs post-I/R, cardiac function (using both echocardiography and hemodynamics) and infarct size were measured. For signaling studies, hearts were harvested at either 15 min post-I/R for preparation of cardiac protein lysates and subsequent analysis as described in the next section. For measurements of cytosolic cytochrome c and BcL-2 proteins (Bad, Bcl-2 and Bcl-xL), 30 min of reperfusion was utilized. Finally, at 3 hrs post-I/R another set of hearts were used for TUNEL staining and caspases-3, -8, and -9 activities for assessment of apoptosis.

### Materials

Antibodies that recognize the either the phosphorylated or non-phosphorylated forms of Akt (Ser473) were purchased from Cell Signaling Technology (Beverly, MA). Antibodies against and Bad, Bcl-2 (sc-7382), Bcl-xL were purchased from Santa Cruz Biotechnology (Santa Cruz, CA). All other chemicals were purchased from Sigma (St. Louis, MO).

### In vivo Cardiac Functional Measurements

We assessed in vivo cardiac function following 24 hrs post-I/R via either echocardiographic measurements (Vevo 770, VisualSonics, Toronto, Canada) or hemodynamic measurements using left ventricular (LV) catheterization (Millar Instruments, Houston, TX) as previously reported [28].

### Determination of LV Infarct Size and Ischemia Area

Myocardial infarct size was determined by Evans blue/TTC double staining as described previously [28], [29].

### Determination of Myocardial Apoptosis

Myocardial apoptosis was determined by TUNEL staining and caspase-3, -8 and -9 activity assays as described previously [29], [30].

### Quantification of Cytochrome C Release

Mitochondrial cytochrome C release was determined as described with some modifications [11], [31]. In brief, 25 mg of myocardial tissue was minced on ice, resuspended in 500 µl of MSH buffer (210 mM mannitol/70 mM sucrose/5 mM HEPES, pH 7.5) supplemented with 1 mM EDTA, and homogenized with a glass dounce homogenizer and teflon pestle. Cytosolic and mitochondrial fractions were separated by differential centrifugation (5 min at 1000 g, 30 min at 17,530 g). The mitochondrial pellet was resuspended in MSH buffer, sonicated for 20 s on ice, and centrifuged at 17,530 g for 30 min at 4°C. The resulting supernatant containing mitochondrial extract from this last centrifuge or cytosolic extract from the first centrifugation (5 min at 1000 g) were separately mixed with Laemmli’s loading buffer, boiled for 5 min, and subjected to SDS/PAGE followed by electroblotting to nitrocellulose. Western blotting for cytochrome C and GAPDH were carried out by standard methods. Results were expressed as the measured cytochrome C level normalized to GAPDH levels as a loading control.

### Neonatal Rat Ventricular Myocytes (NRVM) Isolation and in vitro Models of Oxidative Stress

NRVMs from 1–2 day old rats were isolated as previously described [25] and GRK2 expression was knocked down using anti-GRK2 small interfering (si)RNA (12 nmol together with HiPerfect Transfection Reagent (Qiagen, Valencia, CA), sense: GCUCAGUUUCAUCCUGGAUtt, antisense: AUCCAGGAUGAAACUGAGCtt). Scrambled siRNA was used as control. After 72 hrs NRVMs were treated with chelerythrine (Chele, 10 µM) for 30 min to induce oxidative stress as described previously [32], [33]. Whole cell lysates were prepared and subjected to immunoblotting to measure levels of GRK2, cleaved caspase-3, Akt, Bcl-2, Bcl-xL and GAPDH.

### Statistical Analysis

All values in the text and figures are presented as mean ± SEM of independent experiments from given n-sizes. Statistical significance was determined by one-way or two-way ANOVA followed by the Bonferroni post-hoc test when appropriate. Probabilities of 0.05 or less were considered to be statistically significant.

## Results

### Lowering Cardiomyocyte GRK2 Levels Improves Post-I/R Functional Recovery

**Figure 1:**
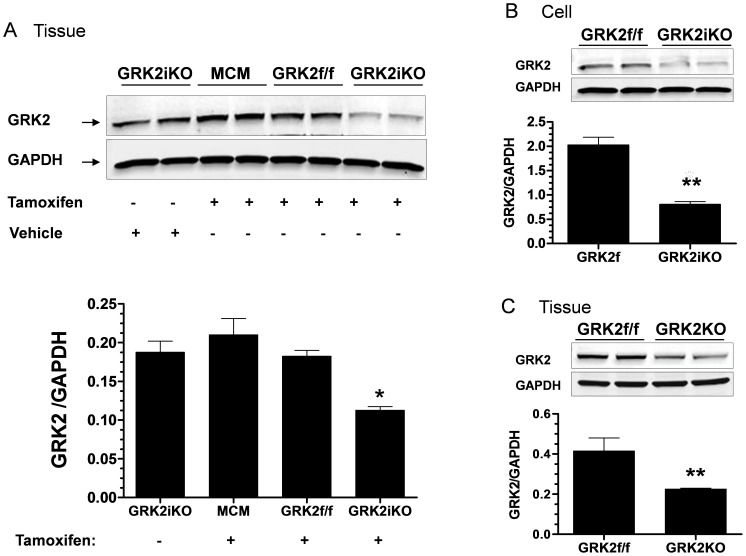
Cardiac GRK2 Expression in Knockout Mice. (**A**) Whole heart GRK2 expression from MerCreMer (MCM), GRK2fl/fl (GRK2f/f) and GRK2iKO (inducible KO) groups before and after the treatment with tamoxifen. * *p*<0.05 vs. GRK2f/f, MCM and GRK2iKO treated with vehicle groups. (**B**) GRK2 expression in cardiomyocytes isolated from GRK2iKO and GRK2f/f control mice. ** *p*<0.05 vs. GRK2f/f. (**C**) Whole heart GRK2 expression from constitutive GRK2KO mice and GRK2fl/fl control mice. ** *p*<0.01 vs. GRK2f/f.

**Figure 2:**
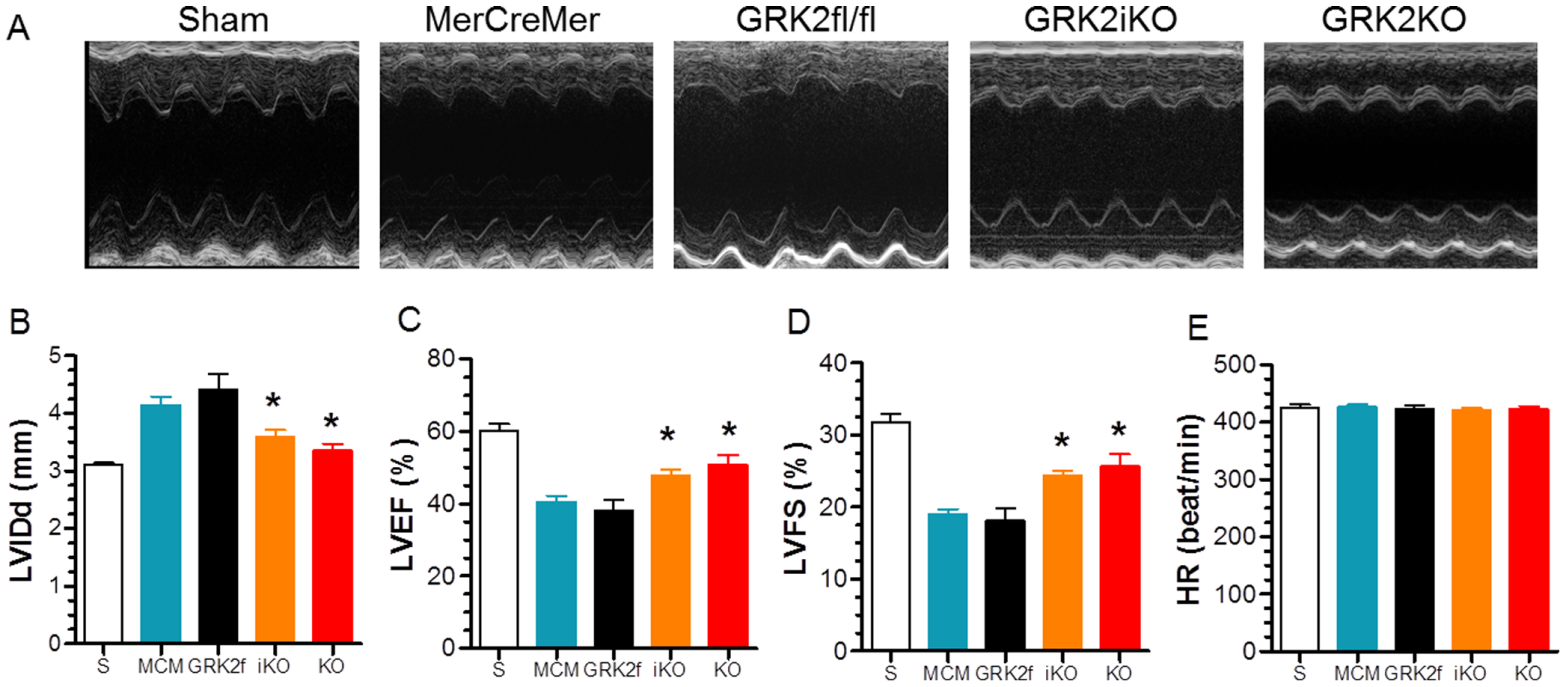
Loss of Cardiac GRK2 Expression Improves Post-I/R Cardiac Function. (**A**) Representative M-mode echocardiography recordings from Sham control mice (S, n = 8) and 24 hr post-I/R mice from different groups (MCM, n = 10, GRK2fl/fl, n = 7, GRK2iKO, n = 14, and GRK2KO, n = 9). (**B-E**) Mean±SEM of measured values from these groups of mice for LV internal diastolic dimension (LVIDd), (**B**); D, LV ejection fraction (LV EF%), (**C**); LV fractional shortening (LV FS%), (**D**); and heart rate (HR), (**E**). * *p*<0.05 vs. MerCreMer (MCM) or GRK2fl/fl control groups.

Studies targeting the role of GRK2 in I/R injury were performed with two independent lines of cardiac-specific GRK2 KO mice: a constitutive GRK2KO line and an inducible GRK2 KO line (termed GRK2iKO) where GRK2 was deleted in adulthood using Tmx. As shown in Fig. 1, significant (≥50%) but not complete deletion of GRK2 in mouse hearts and cardiomyocytes was observed in either line, which is consistent with our previous report [22], [27]. To investigate the impact of lowered cardiomyocyte GRK2 levels on ischemic injury, we first measured in vivo cardiac function using echocardiography and terminal hemodynamics at 24 hrs post-I/R in both lines of GRK2 KO mice along with their respective controls (MCM for GRK2iKO and GRK2fl/fl for GRK2KO). As shown in Fig. 2, both lines of control mice exhibited impaired anterior wall motion post-I/R when compared to sham groups that was significantly improved in GRK2KO and GRK2iKO mice. Of note, sham animals from each line were initially used and since cardiac function was similar amongst them we subsequently combined them into a single sham control group. Specifically, MCM and GRK2fl/fl mice after I/R had significantly increased left ventricle (LV) diastolic (LVIDd) chamber dimensions and accordingly, decreased LV ejection fraction (LVEF) and fractional shortening (LVFS) compared to Sham controls (Fig. 2B–E). However, GRK2 lowering in myocytes led to significantly smaller dimensions and improved function, a result that indicates lower GRK2 levels can improve acute cardiac function post I/R (Fig. 2).

**Figure 3:**
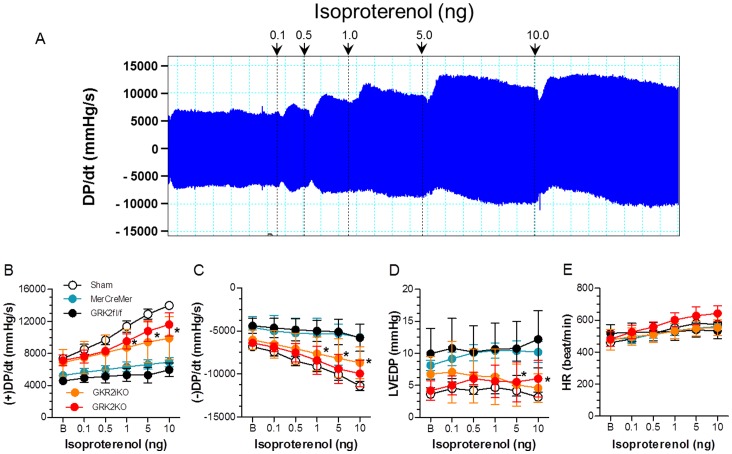
Loss of Cardiac GRK2 Expression Improves Post-I/R Hemodynamic Function. (**A**) Representative original LV dP/dt data tracing acquired from a Sham animal. Data was recorded at baseline (B) and upon isoproterenol treatment (0.1–1.0 ng/mouse). (**B–E**) Hemodynamic data (mean±SEM) recorded B and after cumulative doses of isoproterenol administration in Sham control mice (n = 8) and post-I/R groups (MCM, n = 8, GRK2fl/fl, n = 6, GRK2iKO, n = 9, and GRK2KO, n = 6) at 24 hrs after I/R. Measurements include LV +dP/dt_max_ (**B**); LV -dP/dt_min_ (**C**); LV end diastolic pressure (LVEDP (**D**); and heart rate (HR) (**E**). *p<0.05 GRK2iKO or GRK2 KO vs. MCM and GRK2fl/fl control groups(two-way ANOVA).

Both constitutive and inducible GRK2 KO lines showed similar improvement that was also found in hemodynamic measurements where inotropic reserve was significantly improved in GRK2 KO mice compared to corresponding control mice post-I/R (Fig. 3). Parameters were studied both at baseline and in response to the βAR agonist isoproterenol and included peak LV pressure, LV end-diastolic pressure (LVEDP), heart rate (HR), and LV +dP/dt_max_ and LV −dP/dt_min_ as measures of ventricular contractility and relaxation, respectively (Fig. 3).

### Lowering Levels of Cardiomyocyte GRK2 Reduces Post-I/R Myocardial Injury

**Figure 4:**
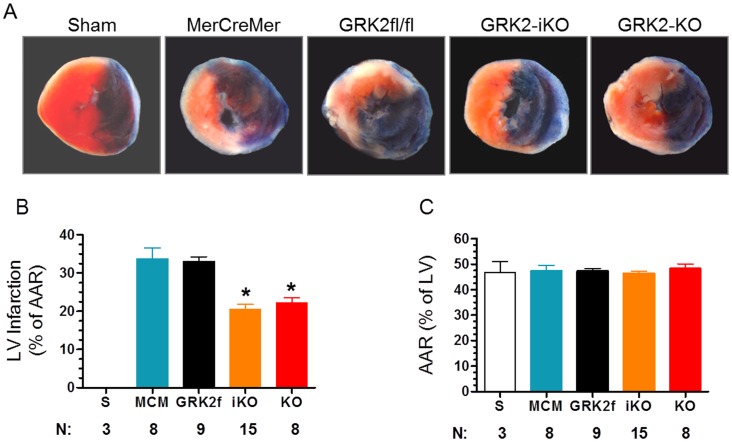
Cardiac GRK2 KO Mice have Reduced Post-I/R Infarct Size. (A) Representative photographs of TTC-stained mouse heart sections obtained from listed groups 24 hrs after a Sham procedure of I/R injury. (**B**) Graphic representation of the LV infarct size expressed as percentage of total ischemic area (Area at risk, AAR) in each group (S, n = 3, MCM, n = 8, GRK2fl/fl, n = 9, GRK2iKO, n = 15, and GRK2KO, n = 8). *p<0.05, vs. MCM and GRK2fl/fl control mice (ANOVA). (**C**) Percentage of LV AAR for each group.

To determine whether reduced GRK2 levels improve cardiac function by directly protecting heart against myocardial I/R injury induced myocyte cell loss we examined post-I/R myocardial infarct size in our two GRK2 KO mice lines. We dissected hearts at the end of 24 hrs of reperfusion and used Evan’s Blue/TTC staining to determine the total LV area at risk (AAR) and infarct size (expressed as % of AAR). Consistent with the in vivo functional data, GRK2iKO and GRK2KO mice had significantly smaller LV infarcts compared to their corresponding control mice (GRK2iKO, 20.6±1.3% of AAR vs. MCM, 33.8±2.9%, *p*<0.05; GRK2KO, 22.29±1.22% vs. GRK2fl/fl, 33.1±1.2%, *p*<0.05) (Fig. 4A and B). This represents a significant ∼35% reduction of infarct size in hearts with GRK2 is reduced to minimal levels in myocytes. Importantly, there was no difference in the measured AAR as a % of the total LV area in any of the mice lines (Fig. 4C).

### Cardiac GRK2 KO Reduces Myocardial Apoptosis after Ischemic Injury

**Figure 5:**
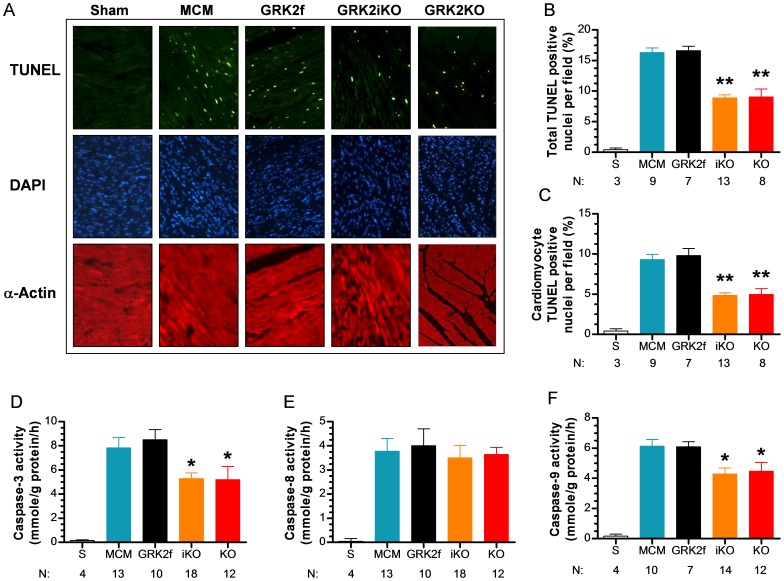
Cardiac GRK2 KO Mice have Reduced Post-I/R Myocardial Apoptosis. (**A**) Representative photomicrographs of in situ detection of DNA fragments (TUNEL) in myocardial sections from Sham or post-I/R mice from the listed groups (MCM and GRK2fl/fl as controls as GRK2iKO and GRK2KO). Total nuclei were labeled with DAPI (blue), and apoptotic nuclei were detected by TUNEL staining (green) and a-actin labeled myocytes in red. (**B,C**) Mean±SEM of the percentage of TUNEL positive nuclei per total cell number per field (**B**) and percentage of TUNEL positive myocytes per field (**C**), *p<0.05 vs. MCM and GRK2fl/fl control groups (ANOVA). (**D–F**) Activity (mean±SEM) of caspase-3 (**D**), -8 (**E**), and -9 (**F**) in all groups with n-sizes per group listed at bottom of graphs, *p<0.05 vs. MCM and GRK2fl/fl control groups.

Strong evidence exists showing that myocardial apoptosis is a major form of cell death following I/R injury. Having demonstrated that lowering GRK2 significantly reduces infarct size, we tested whether there were specific changes in myocardial apoptosis by using TUNEL staining in vivo on cardiac sections and also determining specific caspase activities assays post- I/R. As shown in Fig. 5A and 5B, total TUNEL positive nuclei were significantly reduced in GRK2 KO mice (GRK2iKO, 8.8±0.5%; GRK2KO, 9.06±1.3%) compared to control mice (MCM, 16.3±0.7%; GR2fl/fl, 16.6±0.7%, *p*<0.05). More importantly, as shown in Fig. 5C, we found a significant reduction in myocyte-specific TUNEL labeling when GRK2 was reduced (4.84±0.35% in GRK2iKO mice and 4.97±0.72% in GRK2KO mice) compared to control groups (9.32±0.3% in MCM mice and 8.90±0.89 in GRK2fl/fl mice, *p*<0.05 vs. corresponding GRK2 KO mice). Moreover, tissue caspase-3 and -9 but not -8 activities were also significantly decreased in the GRK2 KO lines compared to control groups after I/R injury (3 hrs) (Fig. 5D–F), suggesting that lowering GRK2 reduces cardiomyocyte apoptosis through mitochondrial-mediated caspase-9 activation (i.e. intrinsic pathway of apoptotic cell death).

### Cardiac GRK2 KO Reduces Mitochondrial Cytochrome C Release after I/R

**Figure 6:**
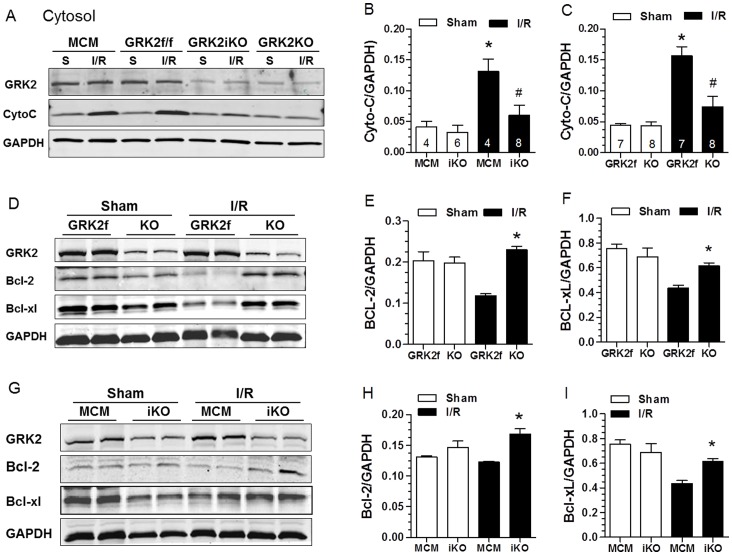
Loss of GRK2 in Cardiomyocytes Decreases Cytochrome C Release from Mitochondria after I/R injury and Increases Levels of the Anti-apoptotic Bcl-2 proteins. (**A**) Representative Western blot of cytosolic levels of GRK2 and cytochrome C (CytoC) in myocardial lysates purified from MCM, GRK2fl/fl, GRK2iKO, and GRK2KO mice 30 min after I/R injury. (**B**) Mean±SEM of CytoC release in MCM and GRK2iKO mice under Sham conditions and post-I/R with n-sizes shown within the individual bars, *p<0.05 MCM-I/R vs MCM-Sham, ^#^ *p*<0.05 GRK2iKO-I/R vs. MCM-I/R mice. (**C**) Mean±SEM of CytoC release in GRK2fl/fl and GRK2KO mice under Sham conditions and post-I/R with n-sizes shown within the individual bars, *p<0.05 GRK2fl/fl-I/R vs GRK2fl/fl-Sham, ^#^ *p*<0.05 GRK2KO-I/R vs. GRK2fl/fl-I/R mice. (**D**) Representative Western blot showing GRK2, Bcl-2 and Bcl-xl protein levels in myocardial lysates purified from Sham or post-I/R GRK2fl/fl control mice or constitutive GRK2KO mice. (**E–F**) Quantitative levels (Mean±SEM) of BCL-2 (**E**) or BCL-xL (**F**) found in myocardial lysates from Sham or post-I/R GRK2fl/fl control mice or GRK2KO mice, *p<0.05 KO vs GRK2fl/fl (n = 4 per group). (**G**) Representative Western blot showing GRK2, Bcl-2 and Bcl-xl protein levels in myocardial lysates purified from Sham or post-I/R MCM control mice or inducible GRK2iKO mice. (H-I) Quantitative levels (Mean±SEM) of BCL-2 (**H**) or BCL-xL (**I**) found in myocardial lysates from Sham or post-I/R MCM control mice or GRK2iKO mice, *p<0.05 KO vs GRK2fl/fl (n = 4 per group).

To obtain more direct evidence that GRK2 may play a key role in mitochondrial-dependent apoptosis of myocytes after I/R injury, we measured cytosolic cytochrome C levels in GRK2 KO and control mice. Cytochrome C is an upstream activator of caspase-9. This is especially important since we did find caspase-9 activity significantly decreased in both lines of cardiac GRK2 KO mice post-I/R (Fig. 5E). As shown in Fig. 6A–C, baseline cytochrome C levels in the cytoplasmic compartment was similar in all groups (GRK2 KO’s and controls), however, in both GRK2KO and GRK2iKO mice there was significantly less cytochrome C released to the cytoplasm after I/R injury (30 min) compared to controls. After I/R, the level of cytochrome C was significantly in control mice increased over 300% while GRK2 KO mice had a significant ∼50% reduction (Fig.6A–C).

### Lowering GRK2 Levels in the Heart Increases Expression of Anti-apoptotic Bcl-2 Proteins and Activated Anti-Apoptotic Kinases

**Figure 7:**
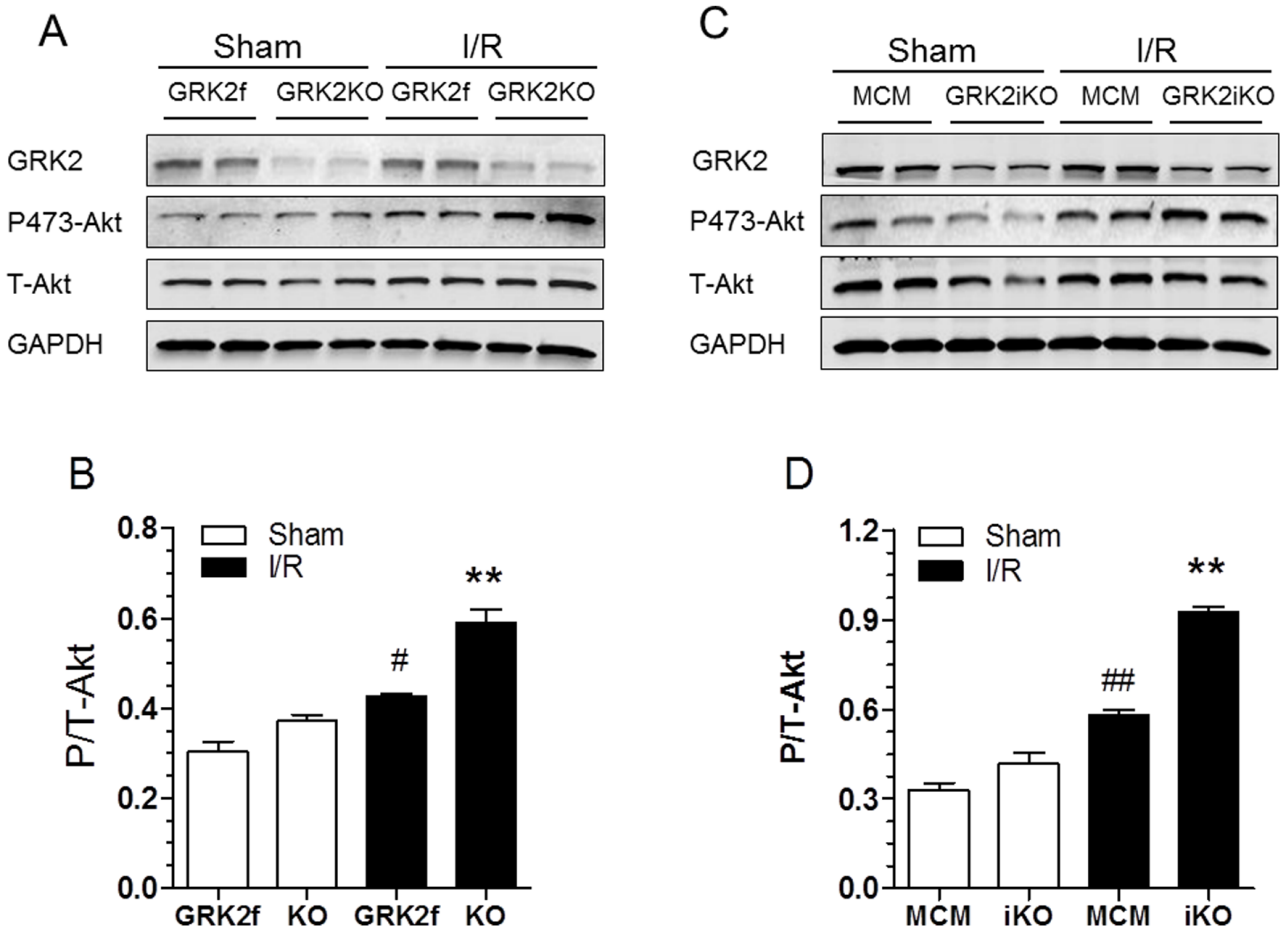
Activated Akt is Increased in Post-I/R GRK2 KO Hearts. (**A**) Representative Western blots for GRK2, total Akt and phospho-Akt (activated via phosphorylation of Ser 473) in myocardial lysates of Sham and post-I/R (30 min I and 15 min R) GRK2fl/fl control mice and constitutive GRK2KO mice. (**B**) Quantitative levels (mean±SEM) of phospho-Akt normalized to total Akt found in myocardial lysates from Sham or post-I/R GRK2fl/fl or GRK2KO mice, *p<0.05 KO vs GRK2fl/fl (n = 4 per group), ^#^ *p*<0.05, GRK2fl/fl-I/R vs. GRK2fl/fl-Sham, **p<0.01, GRK2KO-I/R vs. GRK2fl/fl-I/R and Sham groups (ANOVA, n = 4 pre group). (**C**) Representative Western blots for GRK2, total Akt and phospho-Akt in myocardial lysates of Sham and post-I/R MCM control mice and inducible GRK2KO mice (as in A-B). (**D**) Quantitative levels (mean±SEM) of phospho-Akt normalized to total Akt found in myocardial lysates from Sham or post-I/R MCM or GRK2iKO mice, **p<0.01, MCM-IR vs. MCM sham; ^##^ *p*<0.01, GRK2iKO-I/R vs. MCM-I/R and Sham groups (ANOVA, n = 4 pre group).

To dissect upstream molecular signaling involved in GRK2 activation of the intrinsic apoptosis pathway, several additional experiments were performed. First, we measured Bcl-2 and Bcl-xL (anti-apoptotic) protein levels in hearts subjected to a Sham procedure or I/R injury (30 min of reperfusion). As shown in Fig. 6D–F, baseline levels of Bcl-2 (E) and Bcl-xL (F) protein were not changed between GRK2 KO and control mice. After I/R, the level of Bcl-2 and Bcl-xL were significantly lower in control mice, however levels of Bcl-2 and Bcl-xL were maintained in GRK2KO mice indicating significantly more protective Bcl-2 proteins available after I/R in these mice. These results were essentially identical in GRK2iKO mice after I/R (Fig. 6G–I). This data suggests that the anti-apoptotic effect of lowering GRK2 may be at least partially due to alterations in Bcl-2 signaling, which can regulate cytochrome C release from mitochondria to activate the apoptosome [5]. Next, we measured the levels of phosphorylated Akt (Ser-473), a pro-survival kinase, at baseline (sham conditions) and 15 min post-I/R where we have found peak myocardial Akt activation (data not show). As shown in Fig. 7A–B, phosphorylated (i.e. activated) Akt levels were slightly but significantly elevated in control GRK2fl/fl mice after I/R injury compared to Sham levels. However, there was significantly more p-Akt in GRK2KO mice. This result was mirrored in GRK2iKO mice when compared to their MCM controls (Fig. 7C–7D).

**Figure 8:**
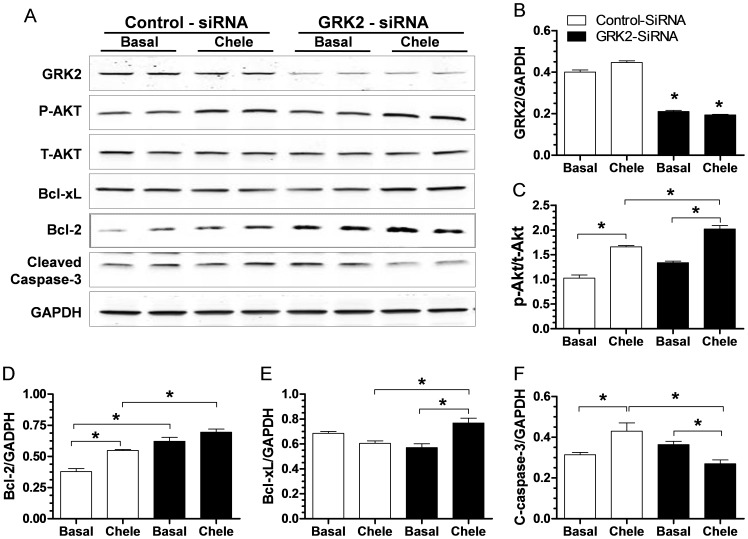
GRK2 Knock-down in Cultured Cardiomyocytes Reduces Cleaved Caspase 3 Levels and Increases Activated Akt and Bcl-2 Levels. (**A**) Representative Western blot for GRK2, total Akt, phospho-Akt (as in Fig. 7), Bcl-2, BCl-xL, cleaved caspase-3 and GAPDH as a loading control cultured neonatal rat ventricular myocytes (NRVMs) following 3 day treatment with control or GRK2-specific siRNA (see Methods) under basal conditions or after a challenge with Chelerythrine (Chele, 10 µM). (**B**) Quantitative assessment of GRK2 levels in NRVMs 3 days after siRNA treatment (control or GRK2 specific siRNA), *p<0.05 GRK2-siRNA vs. Control-siRNA (ANOVA, n = 4 per group). (**C–F**) Quantitative assessment of protein levels of phosphor-Akt normalized to total Akt (**C**); Bcl-2 (**D**); Bcl-xl (**E**); and cleaved caspase-3 (**F**) from the corresponding NRVMs, *p<0.05 between groups as indicated in each graph, n = 4.

To validate our in vivo findings of lowered apoptosis after I/R in GRK2 KO mice, we used a siRNA strategy in neonatal rat ventricular myocytes (NRVMs) to knock-down GRK2 expression and then challenged the cells with chelerythrine (chele), an agent which is known to induce oxidative-stress dependent apoptosis in myocytes [32], [33]. The siRNA treatment decreased GRK2 expression in NRVMs by ≥50% (Fig. 8A–B). As shown in Fig. 8A, and 8F, cleaved caspase-3 was significantly increased in control cells after chele treatment while in cells with lower GRK2 there was significant attenuation of cleaved caspase-3. This blunted response correlated with increased level of phospho-Akt (Fig. 8C), and levels of anti-apoptotic Bcl-2 proteins (Fig. 8D–E). Thus, we found both in vivo and in vitro that myocyte apoptotic signaling after stress can be limited after decreasing GRK2 levels.

## Discussion

In the present study, we directly investigated the mechanistic role of GRK2 as a pro-death kinase in the heart following ischemic injury. We conducted our studies in two independent lines of cardiomyocyte-specific GRK2 KO mice (constitutive and inducible) and found that lower GRK2 levels limits acute ischemic damage after I/R injury. This was manifested by decreased apoptosis, smaller LV infarcts and improved post−/I/R cardiac function. These results support recent evidence by our laboratory showing that overexpression of GRK2 in myocytes enhances ischemic injury and apoptosis. Our results herein are novel as we now have shown how the loss of GRK2 expression can be directly cardioprotective via a significant alteration of pro-survival kinases and apoptotic molecules. Most importantly, we have shown for the first time that with lower myocyte GRK2, there is less cytochrome C release from mitochondria after I/R injury implicating a mitochondrial-dependent apoptotic process. Indeed, activation of caspase-9 was lower after I/R in GRK2 KO mice compared to control mice.

Our data suggests that GRK2 is a nodal regulator of the apoptotic process after ischemic injury coordinating inhibition of pro-survival kinases such as Akt and anti-apoptotic Bcl molecules. Reciprocally, GRK2 promotes cytochrome C release showing that this kinase is a critical regulator of myocyte death. Having the fact of pro-apoptosis in myocytes exposed to oxidative stress, it appears that GRK2 regulates cell death independent of its GRK activity and this is a novel role for this kinase in the heart. These effects of GRK2 on myocyte apoptosis appear to be independent of its GRK activity on GPCRs and support recent data published by us showing that the pro-death effects of GRK2 in myocytes after ischemic and oxidative stress are associated with its unique mitochondrial targeting [33].

GRK2 is up-regulated in the heart acutely after ischemic stress [23], [34]. In fact, it is one of the earliest adrenergic-associated molecular changes after cardiac ischemia [23]. GRK2 expression and activity remains elevated chronically in HF where it promotes the desensitization and down-regulation of βARs causing the loss of inotropic reserve [34], [35]. This has been thought of as a protective mechanism to limit receptors in light of enhanced sympathetic nervous system activity and catecholamine bombardment. However, our data is a direct challenge to this concept as we have shown that the loss of GRK2 expression in myocytes prior to any stress or injury offers significant cardioprotection. Moreover, we have recently shown that the inducible loss of myocyte GRK2 expression after MI–induced HF is already evident, significantly improves cardiac function with active reverse LV remodeling [22].

Our current results from two different GRK2 KO models showing cardioprotection after I/R injury support our previous results where cardiac expression of the GRK2 inhibitory peptide, βARKct, led to significant cardioprotection and lower myocyte apoptosis after ischemic injury [25]. Our results are particularly important since the exact mechanistic target of the βARKct has been questioned due to it targeting the G_βγ_-activation of GRK2 [20]. However, since we find the same post-I/R phenotype it is clear that the βARKct is cardioprotective due to the inhibition of the pro-death effects of GRK2, which is something we also have recently found for the mitochondrial-dependent cell death mechanism and targeting of GRK2 [33], [36], [37]. Our data now indicates that the loss of GRK2 acutely blocks a central role that GRK2 plays in myocyte apoptotic signaling, which appears to be downstream of oxidative stress and independent from its role as a GPCR kinase. Indeed, several intracellular kinase cascades were altered when GRK2 was lowered including the primary pro-survival kinase, Akt. Interestingly, previous data in liver endothelial cells have shown a direct interaction between increased GRK2 expression and Akt leading to its (Akt’s) inhibition [38]. Although, we could not confirm a direct interaction in myocytes (data not shown), our data is consistent with a loss of GRK2 leading directly to increased Akt activation, at least after ischemic injury. Whether this is actually a GPCR-dependent process remains to be addressed. However it is clear that the loss of GRK2 expression can lead to increased Akt activation after I/R injury and this limits ischemic injury in the heart.

It is well documented that Akt improves myocardial survival after I/R injury by inhibiting apoptotic cell death through the phosphorylation of the death agonist Bcl-xL/Bcl-2–associated death promoter (BAD) [15]. Phosphorylation of BAD (at Ser136) by Akt leads to the release of both antiapoptotic Bcl-2 protein Bcl-xL and Bcl-2 [15], [16], which will limit the activation of Bak or Bax, the pro-apoptotic Bcl-2 proteins. We did test the novel hypothesis that this can be controlled by GRK2 after I/R injury and this appears to be the case. Although, we could not detect phosphorylated Bad in our tissue preparation (data not shown), we found that the total level of Bcl-2 and Bcl-xL were increased after I/R in GRK2 KO mice compared to control animals. This increase in the pro-survival Bcl-2 proteins may inhibit the activity of either Bak or Bax and stabilize the mitochondrial membrane transition pore (mPTP) and reduce cytochrome C release, which is one of the novel findings of the study as this mechanism may partially explain the strong anti-apoptotic effect of lowering GRK2. Moreover, as detailed above, other recent studies by us show that the pro-death activity of GRK2 is dependent on its mitochondrial targeting [33] where Akt and these pro- and anti-apoptotic signaling molecules can also be localized.

We have found several mechanisms by which GRK2 lowering might regulate myocyte apoptosis and indicate an unrecognized importance of this kinase in the injured myocyte. It appears that GRK2 lies at a nodal point of regulation for multiple cellular signaling pathways that promotes apoptosis following ischemic injury and oxidative stress and its loss increases cell survival through activation of kinase cascades. This includes signaling mechanisms associated with mitochondria leading to cytochrome C release that can be significantly attenuated post-I/R by lowering GRK2 levels. Overall, our results shed new light on how inhibition or targeting of GRK2 in the heart can be therapeutic including acutely as a cardioprotective agent after ischemic injury.
